# Unannounced standardized patients: a promising method of assessing patient-centered care in your health care system

**DOI:** 10.1186/1472-6963-14-157

**Published:** 2014-04-05

**Authors:** Sondra Zabar, Kathleen Hanley, David Stevens, Jessica Murphy, Angela Burgess, Adina Kalet, Colleen Gillespie

**Affiliations:** 1 Division of General Internal Medicine, Department of Medicine, New York University School of Medicine, 550 First Avenue, New York, NY 10016, USA; 2 NYC Health & Hospitals Corporation, 346 Broadway #5, New York, NY 10013, USA

**Keywords:** Quality improvement, Unannounced standardized patients, Patient-centered care, Assessment, Quality of care

## Abstract

**Background:**

While unannounced standardized patients (USPs) have been used to assess physicians’ clinical skills in the ambulatory setting, they can also provide valuable information on patients’ experience of the health care setting beyond the physician encounter. This paper explores the use of USPs as a methodology for evaluating patient-centered care in the health care system.

**Methods:**

USPs were trained to complete a behaviorally-anchored assessment of core dimensions of patient-centered care delivered within the clinical microsystem, including: 1) Medical assistants’ safe practices, quality of care, and responsiveness to patients; 2) ease of clinic navigation; and 3) the patient-centeredness of care provided by the physician. Descriptive data is provided on these three levels of patient-centeredness within the targeted clinical microsystem. Chi-square analyses were used to signal whether variations by teams within the clinical microsystem were likely to be due to chance or might reflect true differences in patient-centeredness of specific teams.

**Results:**

Sixty USP visits to 11 Primary Care teams were performed over an eight-month period (mean 5 visits/team; range 2–8). No medical assistants reported detecting an USP during the study period. USPs found the clinic easy to navigate and that teams were functioning well in 60% of visits. In 30% to 47% of visits, the physicians could have been more patient-centered. Medical assistants’ patient safety measures were poor: patient identity was confirmed in only 5% of visits and no USPs observed medical assistants wash their hands. Quality of care was relatively high for vital signs (e.g. blood pressure, weight and height), but low for depression screening, occurring in only 15% of visits. In most visits, medical assistants greeted the patient in a timely fashion but took time to fully explain matters in less than half of the visits and rarely introduced themselves. Physicians tried to help patients navigate the system in 62% of visits.

**Conclusions:**

USP assessment captured actionable, critical, behaviorally-specific information on team and system performance in an urban community clinic. This methodology provides unique insight into the patient-centeredness and quality of care in medical settings.

## Background

Newly developed models of health care delivery such as Patient-Centered Medical Homes (PCMH), Accountable Care Organizations (ACO) and explorations of the features of a clinical unit or microsystem that are associated with high quality care [1] are based on an appreciation of the impact of the entire care system on patients’ health. A central principle of these models is that care should be oriented around the needs of patients. Ensuring such patient-centered care requires understanding how patients experience care from the moment they walk in the door until they walk back out, including the range of health care professionals with whom they interact, the functioning of health care teams, the ease with which they can find their way and or get help navigating through the system, and the quality and safety of the services and care they receive throughout that process.

Maximizing health outcomes and patient safety requires a well-designed patient-centered health care system which includes everyone the patient encounters—physicians, nurses, clerks and paraprofessionals. High quality care performed by an individual cannot overcome a poorly run system or team [2]. Poor functioning teams contribute to errors such as increased nosocomial infections or patients not following through on recommended tests [3-5]. Unfortunately, efforts to address dysfunctional teams or clinical units are often driven by isolated incidents or complaints and conducted as post hoc evaluation that does not capture the real time, routine behavior of a clinical system. Proactive initiatives to continuously monitor the quality of the care provided in a clinical system or unit, from the perspective of patients, are critical for improving quality.

Current methods for measuring the functioning of the clinical health system have significant limitations as well as strengths. Direct observation is intrusive and therefore may not reflect every-day, actual functioning; in most contexts, care measured through direct observation is generally assumed to be of much higher quality because providers are aware they are being assessed. However, direct observation’s use of a highly trained observer contributes to its status as one of the most reliable methods for assessing the care that is actually provided [6-9]. Data collected through patient exit interviews have often been shown to be biased in multiple ways, ranging from patients’ reluctance to judge their care negatively [10] to the influence of patients’ prior experience, expectations, health care status/needs, and personality [11]. Patient satisfaction surveys suffer from similar limitations [6,12-14]. However, what both methods may lack in internal validity can be balanced by the generalizability benefits associated with understanding how individuals representative of the targeted patient population respond to and experience health care. This trade-off is especially important when the goal is to understand the impact of health care on patients. When the focus of the assessment is on the practices and processes of the health care system, patient characteristics, on the other hand, can contribute uncontrolled "noise" to the equation, making it difficult to identify how much of health care system responses are due to specific patient variables and how much are truly attributes of the system.

We believe that unannounced standardized patient (USP) visits provide a unique perspective on the functioning of health care systems because they combine a number of methodological strengths: 1) they avoid the "Hawthorne effect" by capturing the practices of health care professionals when they are not aware of being assessed; 2) they involve a highly trained observer/assessor (the USP); 3) they focus on the vantage point of the patient; 4) they control not only for the influence of patient characteristics on recall and evaluation of care through the use of a highly trained professional but also, because they are standardized in terms of the clinical features of the case and the demeanor and personality of the patient, for the effects of such characteristics on the health care system response.

Unannounced standardized patients are actors trained to enter a clinical setting, portray a patient and evaluate performance, traditionally focusing on the patient/provider interaction. USPs have been used as an innovative research method to assess physicians’ compliance with clinical guidelines, the effects of patients’ requests for direct-to-consumer advertised treatments, and residents’ professionalism in an emergency setting [15-17]. The USP method offers great promise for overcoming many of the methodological limitations of traditional methods of assessing the health care system. USPs are trained to portray a clinical condition and a character in a standardized way, controlling for bias associated with how clinical teams may respond to different patients with different needs and characteristics. USPs can be trained to be consistent and accurate raters of clinical performance and clinic functioning through the use of highly specific, behaviorally-anchored checklists [18-20]. They also have the benefit of exposure to a wide variety of levels of performance and training in expectations and standards of quality, experiences most "real" patients do not have. Because of the "unannounced" nature of these visits, behaviors and actions that are captured reflect what really happens in health care systems, i.e. when key personnel are unaware of their evaluation. We have been using USPs to assess physicians’ clinical skills in the Ambulatory clinic and Emergency room setting and have expanded our focus to include the clinical microsystem within which these visits are embedded.

The aims of this study were to explore the feasibility of using the USP methodology to assess the functioning of the patient-centered health system in an inner-city community clinic and to describe what it revealed about patient-centered health system performance.

## Methods

### Setting

USPs were sent into a freestanding city primary care clinic, which generates over 270,000 ambulatory care visits a year. All USPs were registered as brand new patients, with clinic cards and mock demographic and insurance information, and presented to one of 12 patient care teams, each of which included a receptionist and primary care providers, each of whom was assigned to one medical assistant. The primary care providers were residents in a medicine residency program who have their continuity clinic at the ambulatory clinic. Medical assistants in this clinic have diverse backgrounds but all complete a five-hour curriculum and meet regularly as a group to discuss clinic procedures. All medical assistants had been at the clinic for more than two years. USP visits were scheduled to ensure that each resident saw four unique USPs. Care within this clinic was organized into 12 teams and the USPs, based on scheduling, ended up presenting to 11 of the 12 teams.

### Case development and SP training

Actors that resembled the population served in the clinic were hired at 25 dollars per hour to portray common clinical scenarios. We recruited a minimum of four actors per case in order to ensure availability; actor availability proved to be our greatest challenge in scheduling visits. The four cases portrayed new patients: a young patient with an exacerbation of a chronic condition, a patient in need of a physical, a patient seeking health information, and a patient presenting with new systematic complaints. Each standardized patient (SP) was trained for four hours on case portrayal and three hours on using the behaviorally specific checklist to evaluate the clinic and the PCP (Please see Table 1 for checklist items). SPs then completed a practice visit with an attending, which was debriefed by an experienced trainer. For quality control purpose, visits were audio recorded by the USPs, who discreetly carried a digital audio recorder in their purse or pocket. After each visit, SPs also met with the project coordinator to debrief the visit.

**Table 1:** Assessment of patient-centered care by unannounced standardized patients (n = 60 visits)

**Focus**	**Domain**	**Area**	**Item**	**Visits (n = 60)**	
				%	N
	Safety	Identity	Asked name	5%	3
			Asked DOB	5%	3
		Infection control	Washed hands (observed)	0%	0
	Quality of care	Vital signs	Took blood pressure	90%	54
			Weighed	93%	56
			Measured height	77%	46
Medical assistants		Screening	Used PHQ-2	15%	9
	Responsive-ness	Courtesy	Greeted in reasonable time frame	70%	42
			Introduced self	5%	3
			Wore a visible name tag	45%	27
			Was professional	55%	33
			Was friendly	33%	20
		Education	Took time to explain things	47%	28
Clinic	Functioning	Navigation	Somewhat easy to navigate	60%	36
			Very easy to navigate	30%	18
		Team	Team functioned somewhat well	33%	20
			Team functioned very well	62%	37
	Navigation		Helped patient understand how to navigate the system	62%	37
	Patient centeredness		Answered all questions	55%	33
Physician			Took a personal interest	65%	39
			Gave enough information	53%	32
			Encounter did not feel rushed	70%	42

### Assessment of patient-centered care

USPs completed the checklist evaluation form after completing the visit in order to avoid detection. While not the focus of this paper, a behaviorally anchored checklist with evidence of its reliability and validity was used to assess physician communication, history gathering, counseling, and treatment plan/management performance [21-27]. We used this as a model for assessing the patient’s experience of the health care system ("patient-centeredness") and reviewed the patient safety, clinical microsystem, patient-centered medical home literature and conferred with two medical directors at a community and hospital-based ambulatory care clinic to identify domains and specific items to pilot for feasibility and face validity [28-33]. We identified six critical domains to include, as well as the core dimensions of patient-centered care for each domain (see Table 1): 1) Medical assistants’ safe practices (confirming patient identity and washing hands), quality of care (vitals and screening patient education), and responsiveness to patients (greeting the patient, introducing themselves, wearing a name tag, presenting with a professional or friendly manner, and taking time to explain things); 2) the functioning of the clinic (including ease of navigation and team functioning); and 3) the patient-centeredness of the care provided by the physician (e.g., answering all questions, taking a personal interest in the patient, giving sufficient information, and not making the patient feel rushed) along with the degree to which they helped prepare the patient to navigate the system once the encounter was over. We included physicians in order to focus on their role as an important part of the clinical system and therefore tailored our assessment to their support of patient-centeredness. Most items were dichotomous (no/yes), but several items used a 3-point scale (not at all, somewhat, very) including ease of navigation and team functioning.

### Human subjects

Both medical assistants and resident physicians were informed that USPs would visit the clinic at some point in the coming year; the former as a means of conducting quality improvement and the latter as part of their residency program’s assessment of clinical competence and professionalism. The data collected on resident physician performance was included in an NYU Institutional Research Board-approved research registry for educational data wherein residents were asked at orientation for permission to use educational and practice data collected as part of their education and training for research purposes. Resident physician data is only included in this paper for physicians who provided written consent to participate in this medical education research registry (n = 15 representing post graduate year 2 and post graduate year 3 cohorts where 100% of residents consented). USPs did not have access to or record identifiers for the data they collected on the functioning of the clinical microsystem and therefore this Quality Improvement project did not qualify as human subjects research.

### Statistical analysis

We provide the distributions for each item in Table 1, grouped within the levels and broader domains of patient-centered care. In addition, we explored whether patient-centered care varied by team, focusing on the 7 teams that had a minimum of four USP visits. Table 2 provides the percent of visits by team for each of our patient-centered care variables and chi-square (and associated *p*) values are reported to signal when variation is not likely to be due to chance, although results should be interpreted with caution given the small number of visits/team.

**Table 2:** Variation in patient centeredness by team (7 teams with 2 or more visits)

	**Domain**	**Area**	**Item**	**% Visits by team**							**Chi Sq (*p)***
				**1**	**2**	**3**	**4**	**5**	**6**	**7**	
				**N = 7**	**N = 7**	**N = 5**	**N = 6**	**N = 5**	**N = 4**	**N = 8**	
	Safety	Identity	Asked name	0%	0%	0%	0%	0%	0%	0%	N/A
			Asked DOB	0%	0%	0%	0%	0%	0%	0%	N/A
		Infection control	Washed hands (observed)	0%	0%	0%	0%	0%	0%	0%	N/A
	Quality of care	Vital signs	Took blood pressure	100%	86%	100%	100%	100%	100%	88%	Chi Sq = 3.81 (.703)
			Weighed	100%	86%	100%	100%	100%	100%	88%	Chi Sq = 3.81 (.703)
Medical assistants			Measured height	20%	43%	80%	100%	80%	100%	88%	Chi Sq = 11.83 (.057)
		Screening	Used PHQ-2	0%	14%	80%	0%	0%	0%	0%	Chi Sq = 26.20 (.003)
	Respon-siveness	Courtesy	Greeted promptly	86%	57%	60%	100%	60%	75%%	63%	Chi Sq = 4.78 (.573)
			Introduced self	14%	0%	20%	0%	0%	0%	13%	Chi Sq = 3.82 (.701)
			Wore a name tag	57%	43%	60%	50%	40%	75%	25%	Chi Sq = 3.60 (.731)
			Was friendly/prof	100%	71%	100%	100%	100%	100%	75%	Chi Sq = 3.96 (.049)
		Education	Explained things	0%	0%	80%	0%	0%	25%	13%	Chi Sq = 33.93 (.001)
Clinic	Function	Navigation	Very easy to navigate	14%	28%	80%	83%	20%	25%	0%	Chi Sq = 26.02 (.011)
		Team	Functioned very well	43%	14%	80%	83%	40%	100%	38%	Chi Sq = 21.59 (.042)

## Results

Sixty USP encounters were carried out in the clinic between March 2009 and November 2009. USPs presented to 11 of the 12 primary care teams (median 5 visits/team; range: 1 – 8; analyses focused on the 10 teams with at least two visits). Each visit lasted on average 39 minutes (SD = 10) and did not vary significantly by case. No medical assistant (MA) reported detecting a USP during the study period; on average, depending on the visit scenario, 22% of residents reported detecting the USP, although this largely occurred *after* the visit, often because of conversations with fellow residents. Measures of resident performance did not vary significantly by whether visit was detected.

### Clinic

USPs found the clinic difficult to navigate in six (10%) visits, somewhat easy to navigate in 18 (30%) visits, and very easy to navigate in 36 (60%) of the 60 visits. Similar distributions were found for team functioning.

### Medical assistants

Patient safety measures were poor: only three (5%) visits involved confirmations of identity and none of the MA were observed to wash their hands. Quality of care as measured by taking vital signs such as blood pressure, weight, and height was relatively high, with blood pressure assessed in 54 (90%) visits and weight assessed in 56 (93%) visits; however, depression screening (PHQ-2) was low, occurring in only 9 (15%) visits. Responsiveness of the MAs to the patient varied from a low of only three (5%) visits involving the MA introducing his/herself to the patient to a high of 42 (70%) visits including being greeted within a reasonable time frame. MAs took the time to explain things to patients in only 28 (47%) of the visits.

### Physician support of patient-centered care

In 37 (62%) visits, USPs reported that the physicians fully helped them understand how to navigate recommended next steps (e.g., obtaining labs and/or follow-up appointments). Most of the visits involved physicians demonstrating patient-centeredness; however, depending on the item assessing patient-centeredness, in 18 to 28 (30% to 47%) visits, the physicians could have been more patient-centered.

### Variation in patient-centered care by teams

Table 2 provides results by team for the 7 teams with at least 4 USP visits. Results suggest both general strengths in care across teams (e.g., teams generally consistently obtained vitals and were courteous) as well as variation across teams. Only one team appeared to consistently screen for depression and that same team was the only team in which Medical assistants explained things to patients in a majority of visits (Team 3). And in terms of overall functioning, teams varied in how easy it was for the USP to navigate the system (ranging from being easy to navigate in none of the visits to 83% of the visits) and how well the team functioned (in one team only 14% of the visits involved teams rated as functioning well and in another team, the teams in all of the visits were described as functioning).

## Discussion

Our results indicate that an unannounced standard patient (USP) program is a promising method for assessing the functioning of the clinical care system within a health care system. USPs can capture actionable, behaviorally specific aspects of the system that are important to clinical care and difficult to gather with other methodologies. Because of the advantages over other methods, USPs have great potential as part of a set of tools to assess the clinical system and patient-centered care in the new patient centered medical home model. Implementing a USP assessment can help clinical settings move beyond patient satisfaction as a measure of performance and focus more on targeted assessments of the quality of care provided. And while interviews with "real patients" are critical for understanding patient experiences and impact of care, USPs are paid professionals who typically have a great deal of experience in varied health care settings as Standardized Patients, who are required to complete comprehensive training, and who receive ongoing feedback to ensure the accuracy and consistency of their observations and evaluations. Such reliability and validity, combined with the standardization of clinical cases and patient portrayal, is particularly useful for comparing performance over time or across clinical units.

The USP assessment results reported here have already had significant benefits for the institution. Clinic and Residency Program leaders have used these data to focus interventions on actionable items such as improving MAs’ hand washing in front of patients and begin new customer service initiatives, including encouraging staff to wear visible name tags and provide more explanations to patients. This has the potential to both be educational for the team and used as quality improvement data.

USPs, "mystery shoppers", or "secret shoppers" can be viewed as a well-designed audit study [34]. These types of studies provide important objective data that can lead to improvement in patient care processes and outcomes. It can also engender strong feelings from staff and providers. Common concerns focus on utilization of scarce resources, exposure of clinical practices and the ethics of deception. USP visits should be utilized as part of a transparent culture of continuous quality monitoring and improvement. The methodological limitations this method overcomes, including poor recall often found in exit interviews involving "real" patients [35,36] biases associated with personal experiences, expectations, and patients’ tendency to avoid overly negative judgments that contribute to overestimation in patient satisfaction surveys [10,11,14] and Hawthorne-like effects wherein awareness that the quality of care is being assessed actually influences the care provided, can also provide strong and convincing evidence that helps motivate skeptical professionals to change system practices.

USPs are a promising method to assess the quality of the implementation of patient-centered care, including Patient Centered Medical Home (PCMH) models, because USPs provide a highly trained, reliable, standardized perspective on all aspects of the patient experience. Patient-centered care models are based on the premise that it is this sum total of care, not just what happens during the physician visit, that determines health outcomes. To achieve optimal outcomes in ambulatory care, the healthcare team must be a multi-disciplinary group of high performing individuals working together and with patients to monitor and improve clinical measures. Patient safety, clinical prevention, and chronic illness care all require a systematic, proactive approach that makes use of advances in technology, development of system-wide best practices, and patient self-management support. Patients must interact with many professional within a complex system, the right information must get to the right people at the right time, including the patient, and patients must understand and participate in care decisions and then be able to access appropriate services. Our checklist can easily be expanded to capture additional PCMH elements—patient’s ability to make a follow up appointment, have a post-visit question answered, or even capture continuity of care in terms of interaction between interprofessional teams to assess the effectiveness of this rapidly growing model of teamwork and care.

Using USP visits to assess the clinical system is a new practice in the health care community, though this methodology has precedents in the business literature [37,38]. Previous work with USPs has used this method primarily for feasibility studies, assessing physician screening and prescribing practices, or evaluating effectiveness of educational interventions [39]. We have expanded the use of USPs for assessing a large part of patients’ experience of the health care system. However, we have not yet used USPs for assessing the care that happens between visits or for assessing continuity of care across clinical systems. The former we expect, though challenging, can be done but we anticipate greater hurdles in achieving the latter. While we believe our checklist captures aspects of the clinical system essential to patient-centered care, it is not complete. For example, it could be expanded to include the National Patient Safety Standards and to cover additional aspects of the patient experience (outside of the care provided by the core primary care team). Given the challenges USPs face in portraying a realistic patient consistently and then reliably and validly evaluating their experience of the health care system, we recommend spending some time finding the right balance of including critical items for assessment while minimizing USP burden.

While our sample size is small, particularly in terms of number of visits per team, our preliminary data suggest that this method and our checklist do capture variation in team or unit patient-centeredness. Thus, USPs can be used to evaluate functioning at multiple levels—from individual professionals within the system to the collective patient-centeredness of particular teams, clinical units, or other organizational structures within or across health care settings and systems.

Further studies are needed to assess the reliability and cost effectiveness of this new methodology. Reliability may be influenced by actors having repeat visits to the clinical system and finding it easier to navigate. In the clinic room, we’ve been able to use audio recordings to assess the quality of USPs’ performance ratings but it is not practical to do so outside the clinic room (both to protect patient confidentiality and because of the technical challenges of obtaining quality recordings). It may be necessary to re-calibrate USPs and/or recruit and train a cadre of USPs new to particular care settings. Institutions that have standardized patients programs are well poised to expand their range of activities and use USPs to assess the clinical care system. Each visit cost on average $100 once actors are trained and cases and checklists are developed. We do not yet know, however, how these costs and their relative benefits compare to other methods of assessing clinic functioning and patient-centered care.

## Conclusions

Since USPs are fully integrated into the health care system in order to avoid detection, they can provide insight into the entire patient experience—from start (walking in the front door) to finish (walking out the front door)—and therefore the core set of health care system variables that may affect quality of care, patient activation, patient safety, and patient outcomes more generally. Our use of USPs in an urban, safety net primary care clinic helped administrators understand patients’ experience of care and documented important targets for quality improvement. This method can be adapted for use in many different settings to answer critical questions about how the totality of patients’ interactions with health care systems and settings may affect care and therefore holds tremendous promise for forging the historically elusive links between process and patient outcomes.

## Abbreviations

MA: Medical assistant; PC: Primary care; SP: Standardized patient; USP: Unannounced standardized patient.

## Competing interests

The authors declare that they have no competing interests.

## Authors’ contributions

SZ, CG, KH, AK, DS, AB conceived of the study, participated in its design and helped to draft the manuscript; AB and JM implemented study, collected data, and helped draft manuscript, CG performed the statistical analysis. All authors read and approved the final manuscript.

## Authors’ information

SZ is a Co-Director of the Residency Program and Interim Division General Internal Medicine, Co-Director of the Program for Medical Education Innovations and Research and heads up NYU School of Medicine’s Standardized Patient Program. CG is Director of Evaluation and Assessment for the Program for Medical Education Innovations and Research and Director of Curricular Evaluation for the Division of Educational Quality and Analytics at the NYU School of Medicine.

## Pre-publication history

The pre-publication history for this paper can be accessed here:

http://www.biomedcentral.com/1472-6963/14/157/prepub

## References

[B1] Nelson EC Batalden PB Huber TP Mohr JJ Godfrey MM Headrick LA Wasson JH Microsystems in health care: part 1: learning from high-performing front-line clinical units Jt Comm J Qual Improv 2002 28 9 472 493 12216343 10.1016/s1070-3241(02)28051-7

[B2] Grumbach K Bodenheimer T Can health care teams improve primary care practice? JAMA 2004 291 10 1246 1251 10.1001/jama.291.10.1246 15010447

[B3] Kohn LT Corrigan JM Donaldson MS To err is human: building a safer health system 1999 Washington, DC: National Academy Press 25077248

[B4] Baggs JG Ryan SA Phelps CE Richeson JF Johnson JE The association between interdisciplinary collaboration and patient outcomes in a medical intensive care unit Heart Lung 1992 21 1 18 24 1735653

[B5] Young GJ Charns MP Desai K Khuri SF Forbes MG Henderson W Daley J Patterns of coordination and clinical outcomes: a study of surgical services Health Serv Res 1998 33 5I 1211 1236 9865218 PMC1070314

[B6] Hermida J Nicholas DD Blumenfeld SN Comparative validity of three methods for assessment of the quality of primary health care Int J Qual Health Care 1999 11 429 433 10.1093/intqhc/11.5.429 10561036

[B7] Madden JM Quick JD Ross-Degnan D Kaffle KK Undercover careseekers: simulated clients in the study of health provider behavior in developing countries Soc Sci Med 1997 45 1465 1482 10.1016/S0277-9536(97)00076-2 9351137

[B8] Simmons R Elias C The study of client–provider interactions: a review of methodological issues Stud Fam Plann 1994 25 1 17 10.2307/2137985 8209391

[B9] Bessinger RE Bertrand JT Monitoring quality of care in family planning programs: a comparison of observations and client exit interviews Int Fam Plan Perspect 2001 27 63 70 10.2307/2673816

[B10] Carr-Hill RA The measurement of patient satisfaction J Public Health Med 1992 14 3 236 249 1419201

[B11] Like R Zyzanski SJ Patient satisfaction with the clinical encounter: social psychological determinants Soc Sci Med 1987 24 4 351 357 10.1016/0277-9536(87)90153-5 3563565

[B12] Sitzia J Wood N Patient satisfaction: a review of issues and concepts Soc Sci Med 1997 45 12 1829 1843 10.1016/S0277-9536(97)00128-7 9447632

[B13] Avis M Bond M Arthur A Questioning patient satisfaction: an empirical investigation in two outpatient clinics Soc Sci Med 1997 44 1 85 92 10.1016/S0277-9536(96)00140-2

[B14] Brody DS Miller SM Lerman CE Smith DG Lazaro CG Blum MJ The relationship between patients’ satisfaction with their physicians and perceptions about interventions they desired and received Med Care 1989 27 11 1027 1035 10.1097/00005650-198911000-00004 2586185

[B15] Krane NK Anderson D Lazarus CJ Termini M Bowdish B Chauvin S Fonseca V Physician practice behavior and practice guidelines: using unannounced standardized patients to gather data J Gen Intern Med 2009 24 1 53 56 10.1007/s11606-008-0826-3 18975037 PMC2607496

[B16] Kravitz RL Epstein RM Feldman MD Franz CE Azari R Wilkes MS Hinton L Franks P Influence of patients’ requests for direct-to-consumer advertised antidepressants: a randomized controlled trial JAMA 2005 293 16 1995 2002 10.1001/jama.293.16.1995 15855433 PMC3155410

[B17] Zabar S Ark T Gillespie C Hsieh A Kalet A Kachur E Manko J Regan L Can unannounced standardized patients assess professionalism and communication skills in the Emergency Department? Acad Emerg Med 2009 16 9 915 918 10.1111/j.1553-2712.2009.00510.x 19673703

[B18] Peabody JW Luck J Glassman P Dresselhaus TR Lee M Comparison of vignettes, standardized patients, and chart abstraction: a prospective validation study of 3 methods for measuring quality JAMA 2000 283 1715 1722 10.1001/jama.283.13.1715 10755498

[B19] Rehans JJ Gorter S Bokken L Morrison L Unannounced standardized patients in real practice: a systematic literature review Med Educ 2007 41 537 549 10.1111/j.1365-2929.2006.02689.x 17518833

[B20] Luck J Using standardized patients to measure physicians’ practice: validation study using audio recordings BMJ 2002 325 679 687 10.1136/bmj.325.7366.679 12351358 PMC126653

[B21] Yedidia MJ Gillespie CC Kachur E Schwartz MD Ockene J Chepaitis A Snyder CW Lipkin M Lazare A Communications training improved student performance: findings from a controlled 3-school experiment JAMA 2003 290 9 1157 1165 10.1001/jama.290.9.1157 12952997

[B22] Tewksbury LR Gillespie C Paik S Richter RA Kalet AL Quality and quantity of patient contact correlates with performance on a clinical skills exam 2007 Toronto, ON: COMSEP

[B23] Tewksbury LR Gillespie C Richter RA Kalet A Medical students with lowest performance on a clinical skills exam poorly self-assess ability 2006 San Francisco, CA: PAS

[B24] Tewksbury LR Gillespie C Richter RA Kalet A The validity of a comprehensive clinical skills exam 2006 Los Angeles, CA: SGIM National Oral Presentation

[B25] Tewksbury LR Richter RA Gillespie C Chase J Kalet A Communication skills are highly correlated with history content: Findings of a comprehensive clinical skills examination for medical students 2005 PAS

[B26] Hochberg MS Kalet A Zabar S Kachur E Gillespie C Berman RS Can professionalism be taught? Encouraging evidence Am J Surg 2010 199 1 86 93 10.1016/j.amjsurg.2009.10.002 20103071

[B27] Stevens DL King D Laponis R Laponis R Hanley K Zabar S Kalet A Medical students retain pain assessment and management skills long after an experiential curriculum: a controlled study Pain 2009 145 3 319 324 10.1016/j.pain.2009.06.030 19632781

[B28] Nishisaki A Keren R Nadkarni V Does simulation improve patient safety?: self-efficacy, competence, operational performance, and patient safety Anesthesiol Clin 2007 25 2 225 236 10.1016/j.anclin.2007.03.009 17574187

[B29] Battles JB Wilkinson SL Lee SJ Using standardized patients in an objective structured clinical examination as a patient safety tool Qual Saf Health Care 2004 13 SUPPL. 1 i46 i50 15465955 10.1136/qshc.2004.009803 PMC1765798

[B30] Berner ES Houston TK Ray MN Allison JJ Heudebert GR Chatham WW Kennedy JI Jr Glandon GL Norton PA Crawford MA Maisiak RS Improving ambulatory prescribing safety with a handheld decision support system: a randomized controlled trial J Am Med Inform Assoc 2006 13 2 171 179 16357350 10.1197/jamia.M1961 PMC1447547

[B31] Rogers JC The patient-centered medical home movement - promise and peril for family medicine J Am Board Fam Med 2008 21 5 370 374 10.3122/jabfm.2008.05.080142 18772290

[B32] Rosenthal TC The medical home: growing evidence to support a new approach to primary care J Am Board Fam Med 2008 21 5 427 440 10.3122/jabfm.2008.05.070287 18772297

[B33] Sia C Tonniges TF Osterhus E Taba S History of the medical home concept Pediatrics 2004 113 5 II 1473 1478 15121914

[B34] Rhodes K Taking the mystery out of "mystery shopper" studies N Engl J Med 2011 365 6 484 486 10.1056/NEJMp1107779 21793739

[B35] Ward J Sanson-Fisher R Accuracy of patient recall of opportunistic smoking cessation advice in general practice Tob Control 1996 5 2 110 113 10.1136/tc.5.2.110 8910991 PMC1759509

[B36] Pbert L Adam A Quirk M Herbert JR Ockene JK Luippold RS The patient exit interview as an assessment of physician-delivered smoking intervention: a validation study Health Psychol 1999 18 2 183 188 10194054 10.1037//0278-6133.18.2.183

[B37] Fix M, Struyk RJ Clear and convincing evidence: measurement of discrimination in America 1993 Washington, DC: Urban Institute Press

[B38] Siminoff L Rogers H Waller A Harris-Haywood S Esptein R Carrio FB Gliva-McConvey G Longo D The advantages and challenges of unannounced standardized patient methodology to assess healthcare communication Patient Educ Couns 2011 82 3 318 324 10.1016/j.pec.2011.01.021 21316182 PMC3064426

[B39] Rethans J Gorter S Bokken L Morrison L Unannounced standardized patients in real practice: a systematic literature review Med Educ 2007 6 537 549 17518833 10.1111/j.1365-2929.2006.02689.x

